# Deglycosylation of Excretory-Secretory Antigens of the Second-Stage Larvae of *Toxocara cati* Improves Its Efficacy in the Diagnosis of Human Toxocariasis

**DOI:** 10.1155/2023/3024063

**Published:** 2023-07-17

**Authors:** Ali Pouryousef, Bahador Sarkari, Amir Mootabi Alavi, Mostafa Omidian, Fattaneh Mikaeili

**Affiliations:** ^1^Department of Parasitology and Mycology, School of Medicine, Shiraz University of Medical Sciences, Shiraz, Iran; ^2^Department of Pathobiology, School of Veterinary Medicine, Shiraz University, Shiraz, Iran

## Abstract

**Background:**

Toxocariasis is an important zoonotic infection, especially in tropical areas. One of the significant challenges in the serodiagnosis of human toxocariasis is the cross-reaction of *Toxocara* antigens with other parasites due to their relatively similar glycan structures. Removing the glycan structure from *Toxocara* excretory-secretory (TES) antigens may increase the efficacy of these antigens in the diagnosis of toxocariasis. The current study aimed to assess the efficacy of deglycosylated *Toxocara cati *excretory-secretory (dTES) antigens for the serodiagnosis of human toxocariasis.

**Methods:**

*Toxocara* ES antigens were prepared from *T. cati* second-stage larvae and deglycosylated using sodium hydroxide (NaOH). The TES antigens, along with the dTES antigens, were used in an ELISA as well as a western blotting system for the detection of anti-*Toxocara* antibodies. Sera samples collected from 30 confirmed cases of toxocariasis, 30 patients with other diseases, and 30 healthy subjects were evaluated by both systems.

**Results:**

The sensitivity of TES and dTES ELISA for the diagnosis of human toxocariasis was 96.67% (95% CI = 82.78–99.92) and 93.33% (95% CI = 77.93–99.18), respectively, while the specificity of dTES (88.33%; 95% CI = 77.43–95.18) increased significantly compared to the TES (80.00%; 95% CI = 67.67–89.22). The sensitivity of both antigens was 100% (95% CI = 88.43–100) by the western blotting system. Moreover, the specificity of TES and dTES antigens was 95% (95% CI = 86.08–98.96) and 98.33% (95% CI = 91.06–99.96), respectively, when using the western blotting system.

**Conclusion:**

Results of the current study indicate that the chemical removal of the glycan epitopes of *T. cati* ES antigens significantly reduces cross-reactivity rates with other parasitic infections. Considering the findings of the present study, the dTES antigens seem to be suitable antigens for the serodiagnosis of human toxocariasis.

## 1. Introduction

*Toxocara canis* and *Toxocara cati* are two common nematodes in dogs and cats. Toxocariasis, a neglected cosmopolitan infection, is caused by the infective larvae (L2) of *T*. *canis* and *T. cati* [1]. The high infection of *Toxocara* in dogs and cats, as the definitive hosts of the parasite, and the increasing presence of these popular animals close to the human settlements (whether as pets or strays) are the two main facts for the widespread worldwide distribution of toxocariasis [2, 3]. Findings of meta-analysis studies indicate that the seroprevalence trend of toxocariasis is increasing worldwide, especially in tropical countries such as Iran [4–7]. On the other hand, based on a recent meta-analysis study in Iran, the mean prevalence rate of toxocariasis in dogs and cats is estimated at 24.2% and 32.6%, respectively [8].

According to the life cycle of the parasite, the transmission routes of toxocariasis are the accidental ingestion of infective eggs from contaminated soil, vegetables, or the consumption of raw or undercooked meat of paratenic hosts, such as chicken, pigs, and cow, containing encapsulated infective larvae [9–11]. The migration of *Toxocara* larva through the bloodstream to various organs of the human body, including the liver, lungs, retina, and even the central or peripheral nervous system causes failure or dysfunction in the involved organs. Human toxocariasis has been presented as visceral larva migrans (VLM), ocular larva migrans (OLM), neurotoxocariasis, and covert toxocariasis. Covert toxocariasis is the most common form of the disease, with only general clinical or paraclinical manifestations such as hypereosinophilia, hyperbillirubinemia, and pneumonia (e.g., fever, shortness of breath, and cough) [1]. Therefore, the symptoms of toxocariasis are not always so obvious for the final diagnosis.

Accurately diagnosing of toxocariasis is always one of the most difficult issues for clinicians. The best method for diagnosis of toxocariasis is the identification of *Toxocara* larvae in the tissue biopsy, but since this method is extremely invasive, the serological methods such as an enzyme-linked immunosorbent assay (ELISA) and western blotting using *Toxocara* excretory-secretory (TES) antigens, along with clinical symptoms and laboratory findings (hypereosinophilia and high levels of specific IgE), are considered reliable methods for the diagnosis of toxocariasis [12, 13]. The sensitivity and specificity of the serological methods differ due to the type of the antigen used for diagnosis. According to different studies and regardless of the type of antigen used, the range of sensitivity and specificity in the serodiagnosis of human toxocariasis by ELISA has been reported to be between 60% and 100% and 36% and 100%, respectively [1].

Since the currently available assays have cross-reactivity with some helminths that have antigenic components similar to TES antigens, the development of a reliable serological assay with high sensitivity and specificity seems necessary [14–16]. The structure of TES antigens is composed of highly immunogenic glycoproteins that act as the important components in stimulating the host's immune response against the larval stage [17].

The secreted glycoproteins of *Toxocara* larvae are mainly characterized by mucin and lectin structures [18]. The molecular weights of TES glycoprotein fractions range from 15 to 400 kDa, and some of them have already been characterized [12, 19, 20]. The fractions of 32 and 120 kDa are rich in N-glycans and O-glycans, respectively [21–23]. It has also been documented that the fraction of 70 kDa of TES antigens is glycosylated [24].

Analysis of antigenic components of helminths shows that glycoprotein or glycolipid is the predominant antigenic structure in these parasites [25]. Previous studies indicated that the similar epitopes of helminth antigens, such as glycan structures, act as a significant source of cross-reactivity in the serodiagnostic methods, especially among similar phylogenetic parasites [16, 26–31]. One of the main reasons for the false positivity in serodiagnosis of toxocariasis is the similarity of some epitopes of *Toxocara* spp. with other helminths [30, 32]. A study by Roldan et al. [16] showed that deglycosylation of TES antigens could minimize the cross-reactivity with phylogenetically related parasites and subsequently improve the serodiagnosis of toxocariasis, especially in terms of specificity.

As mentioned before, the prevalence of *T. cati* is higher than that of *T. canis* in Iran. On the other hand, the presence of cats as a pet is more prevalent among Iranians. Therefore, it seems that *T. cati* has a more significant role in human toxocariasis, at least in Iran. The present study aimed to evaluate the efficacy of deglycosylated TES (dTES) antigens in the diagnosis of human toxocariasis, using ELISA and western blotting methods.

## 2. Methods

### 2.1. Production of *T. cati *Excretory-Secretory (TES) Antigens

TES antigens were prepared from the *T. cati* second-stage larvae (L2) as described by Zibaei et al. [19] with a few modifications. Briefly, *T. cati* female worms were obtained from the intestine of necropsied stray cats (Shiraz, Iran). The female worms were placed in a 2.5% formalin/ringer solution for approximately four weeks at 25°C until *T. cati* eggs were embryonated. Embryonated eggs were decoated by sodium hypochlorite solution (6–14% active chlorine) at room temperature (RT) for 30 min and then were washed three times with sterile phosphate-buffered saline (PBS) to eliminate the effect of chlorine. After washing, over 90% of the eggs were hatched by an ultrasonic water bath for 5 seconds. The released larvae were collected using a cell strainer (40 *μ*m) under aseptic conditions. The recovered larvae were cultivated in the RPMI 1640 medium (Sigma-Aldrich Chemie GmbH, Germany) containing L-gluta MAX, HEPES 25 mM, sodium bicarbonate, penicillin/streptomycin (100 IU, 100 *μ*g/ml) and amphotericin B (2.5 *μ*g/mL) with 5% CO_2_ at 37°C. The culture supernatant containing the TES antigens was collected and then replaced with fresh medium every week. The TES antigen was dialyzed against distilled water overnight at 4°C. The dialyzed TES antigens were lyophilized and their protein content was determined by the NanoDrop 2000°C spectrophotometer (Thermo Scientific) based on the manufacturer's instructions.

### 2.2. Production of Deglycosylated *T. cati* Excretory-Secretory (dTES) Antigens

Chemical deglycosylation of TES antigens through *β*-elimination was performed according to the method described by Roldan et al. [16]. Briefly, the TES antigens were treated with 100 mM sodium hydroxide (NaOH) at 37°C for 4 h. After that, the reaction was neutralized by adding an equimolar concentration of hydrochloric acid (HCl). The chemical deglycosylation procedure was investigated using carbohydrate and protein staining methods, followed by sodium dodecyl sulfate-polyacrylamide gel electrophoresis (SDS-PAGE) of dTES antigens.

### 2.3. Sodium Dodecyl Sulfate-Polyacrylamide Gel Electrophoresis (SDS-PAGE)

The dTES antigens were separated according to molecular weight using SDS-PAGE on a 15% (w/v) polyacrylamide gel. In brief, dTES antigens were diluted in 2X sample buffer (1% 2-mercaptoethanol, 1% SDS, 16% 1 M Tris pH 6.8, 20% glycerol, and 0.2% bromophenol blue) and incubated at 100°C for 10 minutes. The prepared antigens were separated by SDS-PAGE using a Mini-PROTEAN III apparatus (Bio-Rad Laboratories, Hercules, CA). The samples were run at a voltage of 60 V for 30 min and then with a constant voltage of 90 V until the tracking dye (bromophenol blue) reached the bottom of the gel. A marker with known molecular weights ranging from 10 to 245 kDa (SinaClon, Iran) was also used in each run.

### 2.4. Carbohydrate and Protein Staining

To analyze the protein and carbohydrate structures in the dTES antigens, SDS-PAGE gels were stained with Coomassie Brilliant Blue R-250 and periodic acid-Schiff (PAS), respectively. The Coomassie blue staining was performed for the detection of protein based on the procedure of Wang et al. [33] with a few modifications. The polyacrylamide gel was fixed in a fixing solution (10% v/v acetic acid, 10% v/v methanol, and 40% v/v ethanol) for 1 h at 4°C. The fixed gel was immersed in a stainless-steel tank containing the staining solution (0.125% Coomassie Brilliant Blue R-250, 5% acetic acid, and 45% ethanol) and gently shaken for about 4 h at RT in the dark. Finally, the gel was put into the destaining solution (5% acetic acid and 45% ethanol) for 1 h. Then it was replaced with the same fresh solution every hour until the background of the gel was destained.

The periodic acid-Schiff (PAS) staining was applied to ensure the absence of carbohydrate residuals in the dTES antigens according to the method described by Roldan et al. [16]. In brief, the gel fixation was performed using 25% isopropanol and 10% acetic acid overnight at 4°C. After washing with 7.5% acetic acid for 15 min, the polyacrylamide gel was soaked in 4% periodic acid for 1 h at 4°C, and then put into the undiluted Schiff reagent for 1 h at 4°C in darkness. The gel was immersed three times in 0.5% potassium metabisulphite for 10 min and washed with 7.5% acetic acid.

### 2.5. Serum Samples

Thirty serum samples were collected from toxocariasis patients, diagnosed based on the serological method using the commercial ELISA kit (NOVALISA™ *T. canis* IgG, NOVATEC Immundiagnostica GmbH, Germany). Moreover, 60 serum samples were obtained as controls from 30 healthy individuals and 30 patients with other diseases, including ascariasis (*n* = 3), strongyloidiasis (*n* = 4), trichostrongylosis (*n* = 3), hydatid cyst (*n* = 4), fascioliasis (*n* = 4), visceral leishmaniasis (*n* = 3), toxoplasmosis (*n* = 3), malaria (*n* = 3), and patients with fever of unknown origin (FUO) (*n* = 3). All serum samples were tested using the commercial ELISA kit to confirm that the control samples were negative. In addition, the samples of other diseases were confirmed by at least one of the following methods: stool examination (ascariasis, strongyloidiasis, trichostrongylosis, and fascioliasis), blood smear (malaria), and serological tests (visceral leishmaniasis and toxoplasmosis). The hydatid cyst patients were diagnosed by the combination of two methods, computed tomography scan and a serological assay. FUO patients were also confirmed by clinical and paraclinical findings.

### 2.6. Enzyme-Linked Immunosorbent Assay (ELISA)

The serum samples were evaluated for the detection of anti-*Toxocara* IgG antibodies by ELISA, using TES and dTES antigens [34]. The 96-well flat-bottom microplates (Nunc, Roskilde, Denmark) were coated with 5 *μ*g/ml of TES or dTES antigens (100 *μ*l/well) in 0.05 M carbonate-bicarbonate buffer (coating buffer, pH 9.6) and incubated overnight at 4°C. The plates were washed three times with PBS containing 0.05% Tween 20 (PBST) (washing buffer); after, each of the wells was blocked with 3% skimmed milk in PBST (blocking solution) and incubated for 2 h at RT. The plates were then washed as before, and diluted serum samples (1 : 100 in PBST) were added to each well (100 *μ*l/well) and incubated for 1 h at RT. Then, horseradish peroxidase-conjugated antihuman IgG (Sigma-Poole, UK) at 1 : 4000 dilution in PBST was added to the wells and incubated for 1 h at 37°C. After washing the plates three times, a chromogen/substrate (0.4 mg/ml OPD (o-phenylenediamine dihydrochloride), 0.3% H_2_O_2_ in 0.1 M citrate buffer, pH 5.6) was added to the plate. Then, the enzymatic reaction was stopped, after 30 min, by the addition of 1 M sulfuric acid (100 *μ*l). Finally, the optical density (OD) of serum samples was determined at a wavelength of 492 nm using an automatic microplate reader (ELX800, BioTek, USA). The cut-off value was calculated by the mean OD of the negative controls plus two standard deviations. To ensure the experiment results, all sera were tested twice.

### 2.7. Western Blotting

After separating the protein fractions of TES and dTES antigens by SDS-PAGE, the proteins were transferred to a nitrocellulose (NC) membrane (Schleicher and Schuell BioScience, Germany) for western blotting, using a constant voltage of 30 V overnight at 4°C. Then, the transfer of the protein fractions from the acrylamide gel to NC was confirmed by Ponceau S staining [19]. The NC membranes containing the protein fractions were cut into strips with the same width (3 mm) and blocked with 5% skimmed milk in PBST for 2 h. The strips were washed three times (each for 5 min) with PBST and then incubated with diluted serum samples (1 : 100 in blocking buffer) for 2 h. After washing as before, horseradish peroxidase-conjugated antihuman IgG (at 1 : 4000 dilution in PBST) was added to each strip and incubated for 2 h. The NC strips were washed three times in PBST and incubated with diaminobenzidine tetrahydrochloride (DAB) chromogenic substrate prepared in 50 mM Tris-HCl, pH 7.6 in the presence of 0.1% H_2_O_2_ for 5 min. Finally, the enzymatic reaction was stopped using tap water. The serum samples of toxocariasis as positive control and healthy person as negative control, along with a standard protein molecular weight marker, were used in each run.

### 2.8. Statistical Analysis

The ROC (receiver operating characteristic) curve analysis was applied to evaluate the sensitivity and specificity of TES and dTES antigens in the detection of anti-*Toxocara* spp. IgG antibodies. The Kappa index was also used to assess the diagnostic agreement between the tests. All statistical analyses were performed using SPSS software (ver. 20), and a value of less than 0.05 was considered statistically significant.

### 2.9. Ethical Considerations

Ethical approval was obtained from the ethics committee of the Shiraz University of Medical Sciences (code: IR.SUMS.REC.1400.240). All participants completed and signed the informed consent form before being involved in the study.

## 3. Results

### 3.1. Chemical Deglycosylation of TES Antigens

**Figure 1:**
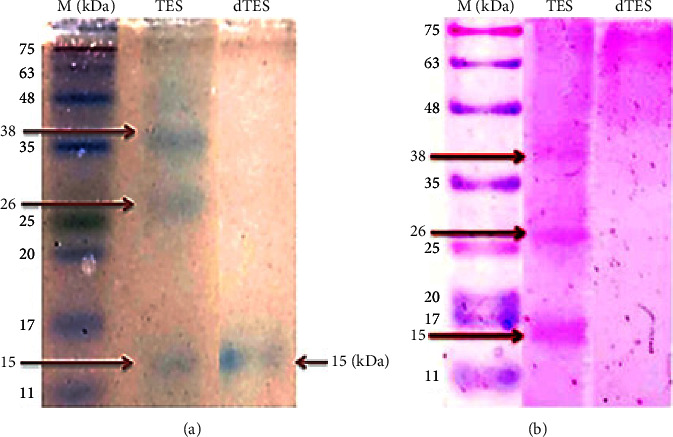
Separation of *Toxocara cati *excretory-secretory (TES) antigens and deglycosylated *Toxocara cati *excretory-secretory (dTES) antigens on a 15% (w/v) polyacrylamide gel using SDS-PAGE, and staining with Coomassie Brilliant Blue R-250 for detection of protein structures (a) and periodic acid-Schiff for detection of carbohydrate structures (b). M: protein molecular weight marker (kDa).

Three fractions with low molecular weight, including 15, 26, and 38 kDa, were detected from TES antigens by SDS-PAGE using 15% gel and Coomassie blue staining (Figure 1(a)). After deglycosylation of the TES antigens with 100 Mm NaOH for 4 h at 37°C, the dTES antigens revealed only one protein band with a molecular weight of 15 kDa (Figure 1(a)).

After treatment of the TES antigens with NaOH in the mentioned condition, the PAS staining of SDS-PAGE gel showed that the carbohydrate structure of the TES antigens had been removed successfully. Therefore, bands at the dTES lane were diminished (Figure 1(b)).

### 3.2. ELISA

**Figure 2:**
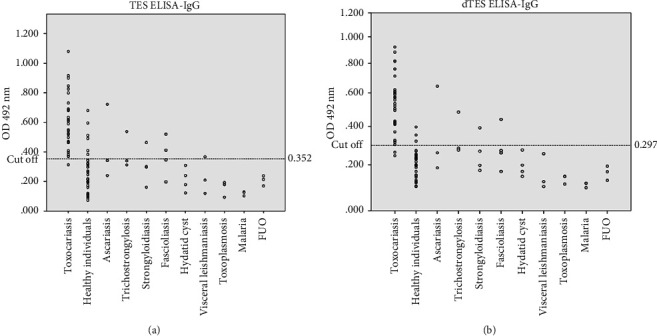
Optical density (OD) of serum samples evaluated by ELISA using *Toxocara cati *excretory-secretory (TES) antigens (a) and deglycosylated *Toxocara cati *excretory-secretory (dTES) antigens (b).

Out of 30 serologically confirmed toxocariasis cases, the ELISA using TES and dTES antigens was positive in 29 (96.67%) and 28 (93.33%) patients, respectively (Figure 2).

Out of 60 control serum samples, TES antigens had the false positive reaction in 12 cases and dTES antigens in seven cases (Figure 2).  Table 1 shows the results of ELISAd using TES and dTES antigens, and Table 2 shows the sensitivity, specificity, positive predictive value, and negative predictive value of ELISA using TES and dTES antigens in the diagnosis of toxocariasis.

The kappa agreement rate of 0.864 was estimated between ELISAs using TES and dTES antigens, and an almost perfect agreement was seen. In addition, the kappa agreement coefficient between the ELISA of TES and dTES compared to the commercial ELISA kit was obtained 0.702 and 0.807, respectively.

**Figure 3:**
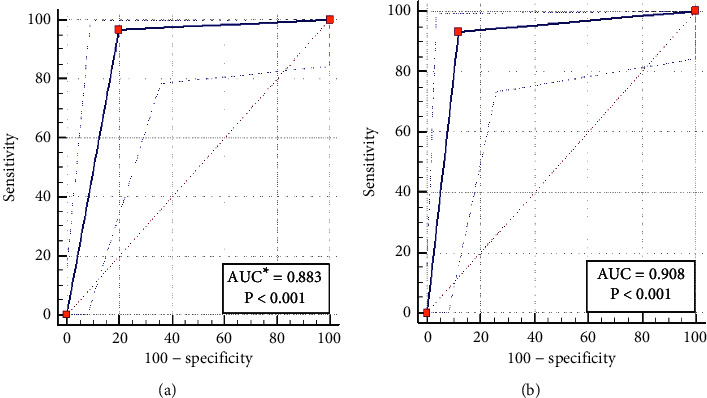
Receiver operating characteristics (ROC) curves for evaluation of sensitivity and specificity of ELISA using *Toxocara cati *excretory-secretory (TES) antigens (a) and deglycosylated *Toxocara cati *excretory-secretory (dTES) antigens (b). ^*∗*^AUC: area under the ROC curve.

The ROC curve analysis of ELISA using TES and dTES antigens among assayed serum samples showed an area of 0.883 (95% CI = 0.798–0.941) and 0.908 (95% CI = 0.829–0.959), respectively (Figure 3).

### 3.3. Western Blotting

**Figure 4:**
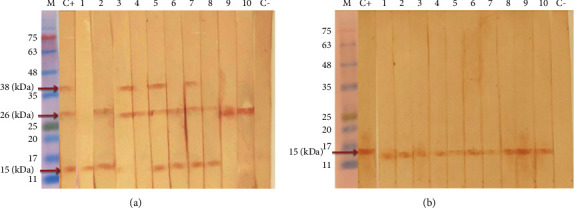
Western blotting using *Toxocara cati *excretory-secretory (TES) antigens (a) and deglycosylated *Toxocara cati *excretory-secretory (dTES) antigens (b). Serum samples of patients with toxocariasis (1–10), positive (C+), and negative (C−) control. M: protein molecular weight marker.

Each of the 30 serologically confirmed toxocariasis cases reacted with at least one of the three fractions of TES antigens: 15 kDa = 21 (70%), 26 kDa = 25 (83.3%), and 38 kDa = 10 (33.3%) (Figure 4(a)). All of the sera from 30 toxocariasis patients reacted against the single fraction of dTES antigen (15 kDa) in the western blotting (Figure 4(b)).

**Figure 5:**
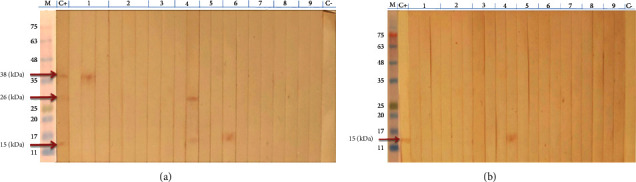
Western blotting using *Toxocara cati *excretory-secretory (TES) antigens (a) and deglycosylated *Toxocara cati *excretory-secretory (dTES) antigens (b). Serum samples of patients with fascioliasis (1), strongyloidiasis (2), hydatid cyst (3), ascariasis (4), visceral leishmaniasis (5), trichostrongylosis (6), toxoplasmosis (7), malaria (8), fever of unknown origin (9), positive (C+), and negative (C−) control. M: protein molecular weight marker.

Samples from healthy subjects did not yield any reaction with the fractions of TES and dTES antigens (Table 1). Western blotting using TES antigens was carried out with sera of 30 patients with other diseases, and reactivity was seen in 3 serum samples, including ascariasis (with 15 and 26 kDa fractions), trichostrongylosis (with 15 kDa fraction), and fascioliasis (with 38 kDa fraction) (Figure 5(a)). However, the specificity of western blotting using dTES antigens was significantly improved, and only one serum sample of ascariasis patients reacted against the single fraction (15 kDa) of dTES antigen (Figure 5(b)). In Table 2, the sensitivity, specificity, positive predictive value, and negative predictive value of western blotting using TES and dTES antigens were shown.

When the results of western blotting using TES antigens were compared with those of western blotting using dTES antigens, a high degree of agreement was observed (kappa = 0.952). Kappa analysis on the results of western blot showed an acceptable agreement of 0.902 for TES and 0.924 for dTES antigens compared to the commercial ELISA kit.

## 4. Discussion

Diagnosis of human toxocariasis is based on the detection of anti-*Toxocara* antibodies by serological methods such as ELISA and western blotting [12, 35]. The main antigens currently employed for the diagnosis of human toxocariasis are TES antigens, but the glycan structures of TES antigens cause cross-reactivity in the serodiagnosis of human toxocariasis [16, 26, 31]. This cross-reaction is problematic in regions where other parasitic diseases are endemic. It has been documented that cross-reactivity in the serodiagnosis of parasitic infections is attributed to reactivity with glycan structures shared among the parasite's antigens [36, 37]. Chemical deglycosylation of antigens through *β*-elimination is a method that can eliminate such glycan structures and may improve specificity in the diagnosis of parasitic diseases. Lorenzo et al. [27] reported that the glycan structures of *Anisakis* simplex antigens are the source of cross-reactivity, and this cross-reaction can be reduced by *β*-elimination of O-glycans from these antigens.

In this study, three fractions of ES antigens of *T. cati* (15, 26, and 38 kDa) were identified by SDS-PAGE. Zibaei et al. [19] detected a range of 20 to 150 kDa fractions of ES antigens of *T. cati* by SDS-PAGE. In the study of Rolden et al. [16], five fractions of ES antigens of *T. canis* (32, 45, 55, 70, and 120 kDa) were indicated by SDS-PAGE. In general, the differences in the detected fractions of TES antigens by SDS-PAGE may be due to the species of *Toxocara* (*T. cati* and *T. canis*), the developmental stage of the parasite (egg, larva, and adult), the preparation conditions of the antigen, and the type of antigen (ES and crude).

Deglycosylation can be done by two methods: enzymatic or chemical method. Enzymatic deglycosylation is the most common method of removing glycan structures, but the high cost of used enzymes is the major disadvantage of this method [38]. Whereas, chemical deglycosylation is a simpler and cheaper method and NaOH is one of the most effective used chemical reagents that release O-glycans and N-glycans from the glycoproteins at different concentrations [16, 39]. Therefore, chemical deglycosylation of TES antigens is simpler, efficient, and practical. In the current study, chemical deglycosylation of ES antigens of *T. cati* second-stage larvae was performed according to Rolden et al. [16], who investigated the deglycosylation of TES antigens with NaOH at different concentrations, temperatures, and times. The best conditions for deglycosylation were obtained with 100 mM NaOH for 4 h at a temperature of 37°C. In our study, after deglycosylation of the TES antigens in the mentioned conditions, no glycan structures were detected by PAS staining. Among the three detected fractions of TES antigens (15, 26, and 38 kDa) by SDS-PAGE, only a protein structure with a molecular weight of 15 kDa was identified after chemical deglycosylation of the TES antigens, whereas, Rolden et al. [16] detected a fraction of approximately 26 kDa. The detection of fractions with a smaller molecular weight after deglycosylation is probably due to the hydrolysis of fractions with a higher molecular weight or their fragmentation into smaller subunits. Page and Maizels [40] detected a polypeptide with a molecular weight of about 15 kDa after the deglycosylation of the 120 kDa fraction of TES antigens. Lorenzo et al. [27] reported that the degradation rate of the protein core during treatment with NaOH mainly depends on the nature of the glycoprotein. In the present study, the 15 kDa fraction of dTES antigens was not characterized, so it is not known that this fraction is the same as the 15 kDa fraction of TES antigens. However, the improvement of specificity of the western blotting method after the deglycosylation process indicates that the obtained fraction with a molecular weight of 15 kDa can be a residual component of other hydrolyzed glycoproteins in this study.

In this study, we evaluated the usefulness of ELISA using dTES antigens for the diagnosis of toxocariasis and compared the results with ELISA using TES antigens. Our findings showed that the dTES-based ELISA was not able to detect the anti-*Toxocara* antibodies in the serum of two toxocariasis patients, which means the elimination of the glycan structures of TES antigens reduced the sensitivity of the dTES-based ELISA compared to the TES-based ELISA. However, when ELISA was performed on the serum samples of healthy controls and patients with other diseases, high specificity was seen for the dTES-based ELISA compared to the TES-based ELISA. Three samples from the healthy controls and four samples from patients with other diseases (ascariasis, trichostrongylosis, strongyloidiasis, and fascioliasis) gave a positive reaction by the ELISA using the dTES antigens, and overall, the dTES-based ELISA demonstrated satisfactory specificity compared to the TES-based ELISA for the diagnosis of human toxocariasis. In the study by Roldan et al. [16], the sensitivity of ELISA using the TES and dTES antigens was 100%, while the specificity of dTES-based ELISA (97.7%) was significantly higher than TES-based ELISA (94.1%). Generally, it is expected that the specificity of ELISA using dTES antigens will increase due to the elimination of glycan structures that cause cross-reactivity. The results showed that the dTES antigens provide higher specificity for the serodiagnosis of toxocariasis. Variation in sensitivity and specificity of ELISA can be justified by the differences in *Toxocara* species used for producing TES antigens, as well as the type and number of examined sera.

ELISA is a standard and common method for the serodiagnosis of human toxocariasis, but western blotting can be considered a confirmatory method due to its higher sensitivity and specificity [12]. Therefore, western blotting is a better method for diagnosis of toxocariasis compared to ELISA, since specific proteins are separated from a mixture of proteins in the western blotting method, and the ability of antibody detection increases by the specific antigens [41]. In this study, as expected, the sensitivity and specificity of western blotting increased for both antigens (TES and dTES) compared to ELISA. We found three fractions (15, 26, and 38 kDa) of TES antigens as the immunodominant proteins, with 30 toxocariasis cases reacting with at least one fraction. In addition, all sera of toxocariasis patients reacted against a single fraction with a molecular weight of 15 kDa of dTES antigen. Therefore, TES and dTES antigens were able to detect anti-*Toxocara* spp. antibodies in all sera of toxocariasis patients, and the sensitivity of western blotting using both antigens was 100%. Different studies have shown that western blotting decreases the cross-reactivity and thus increases the specificity in the serodiagnosis of human toxocariasis compared to ELISA [41]. We performed western blotting using TES and dTES antigens on sera from patients with other parasitic infections, such as ascariasis, strongyloidiasis, trichostrongylosis, hydatid cyst, fascioliasis, visceral leishmaniasis, toxoplasmosis, and malaria, for evaluation of the specificity of TES and dTES antigens. Sera from patients with ascariasis and trichostrongylosis reacted with the 15 kDa fraction of TES antigens. However, only one serum from patients with ascariasis was reacted with the single fraction of dTES antigen (15 kDa), and the specificity of western blotting using the dTES antigens was significantly improved. Although fractions with high molecular weight were not identified in this study, fractions with low molecular weight (15, 26, and 38 kDa) had acceptable diagnostic performance. In agreement with our results, several studies reported that fractions of *Toxocara* spp. ES antigens with low molecular weight are more specific and are often not recognized by nonspecific IgG antibodies [13, 16, 19, 42, 43].

The study by Watthanakulpanich et al. [44] regarding the performance of IgG subclass antibodies (IgG1-4) in serodiagnostic assays of human toxocariasis showed that the IgG3 subclass was more specific in detecting the TES antigens than other subclasses, and reduces the false positivity. The IgG2 subclass gave the greatest sensitivity, since it recognises carbohydrate epitopes of TES antigens. Considering the study mentioned above, the detection of anti-*Toxocara* IgG3 antibodies using dTES antigen may reduce the cross-reactivity rate and improve the test specificity.

## 5. Conclusion

To the best of our knowledge, this is the first report on the use of deglycosylated *Toxocara cati *excretory-secretory (dTES) antigens in the serodiagnosis of toxocariasis. Our study demonstrated that the elimination of glycosidic components, as epitopes responsible for cross-reactivity, improves the specificity of ELISA and western blotting systems for the serodiagnosis of human toxocariasis. The use of dTES antigens with a larger sample size and more various helminth infections is suggested for further confirmation of the findings of this study. In addition, the simultaneous use of deglycosylated ES antigens of *T. canis* and *T. cati* may improve the diagnostic efficacy of ELISA or western blotting for the serodiagnosis of human toxocariasis.

## Figures and Tables

**Table 1:** Results of ELISA and western blotting using *Toxocara *excretory-secretory (TES) and deglycosylated *Toxocara *excretory-secretory (dTES) antigens on 30 confirmed cases of toxocariasis, 30 patients with other diseases, and 30 healthy individuals for the serodiagnosis of human toxocariasis.

Samples		Toxocariasis	HI	Other diseases								
				A	S	Tri	H	F	VL	T	M	FUO
No. of cases		30	30	3	4	3	4	4	3	3	3	3
TES ELISA	**+**	29 (96.7%)	6 (20%)	1 (33.3%)	1 (25%)	1 (33.3%)	0 (0%)	2 (50%)	1 (33.3%)	0 (0%)	0 (0%)	0 (0%)
	**−**	1 (3.3%)	24 (80%)	2 (66.7%)	3 (75%)	2 (66.7%)	4 (100%)	2 (50%)	2 (66.7%)	3 (100%)	3 (100%)	3 (100%)

dTES ELISA	**+**	28 (93.3%)	3 (10%)	1 (33.3%)	1 (25%)	1 (33.3%)	0 (0%)	1 (25%)	0 (0%)	0 (0%)	0 (0%)	0 (0%)
	**−**	2 (6.7%)	27 (90%)	2 (66.7%)	3 (75%)	2 (66.7%)	4 (100%)	3 (75%)	3 (100%)	3 (100%)	3 (100%)	3 (100%)

TES western blotting	**+**	30 (100%)	0 (0%)	1 (33.3%)	0 (0%)	1 (33.3%)	0 (0%)	1 (33.3%)	0 (0%)	0 (0%)	0 (0%)	0 (0%)
	**−**	0 (0%)	30 (100%)	2 (66.7%)	4 (100%)	2 (66.7%)	4 (100%)	3 (66.7%)	3 (100%)	3 (100%)	3 (100%)	3 (100%)

dTES western blotting	**+**	30 (100%)	0 (0%)	1 (33.3%)	0 (0%)	0 (0%)	0 (0%)	0 (0%)	0 (0%)	0 (0%)	0 (0%)	0 (0%)
	**−**	0 (0%)	30 (100%)	2 (66.7%)	4 (100%)	3 (100%)	4 (100%)	4 (100%)	3 (100%)	3 (100%)	3 (100%)	3 (100%)

HI = healthy individuals, *A* = ascariasis, S = strongyloidiasis, Tri = trichostrongylosis, H = hydatid cyst, F = fascioliasis, VL = visceral leishmaniasis, *T* = toxoplasmosis, *M* = malaria, FUO = fever of unknown origin.

**Table 2:** Sensitivity, specificity, positive predictive value, and negative predictive value of ELISA and western blotting using *Toxocara *excretory-secretory (TES) and deglycosylated *Toxocara *excretory-secretory (dTES) antigens for serodiagnosis of human toxocariasis.

Assay	ELISA using TES antigens	ELISA using dTES antigens	Western blotting using TES antigens	Western blotting using dTES antigens
Sensitivity (95% CI)	96.67% (82.78–99.92)	93.33% (77.93–99.18)	100% (88.43–100)	100% (88.43–100)
Specificity (95% CI)	80.00% (67.67–89.22)	88.33% (77.43–95.18)	95.00% (86.08–98.96)	98.33% (91.06–99.96)
PPV^1^ (95% CI)	70.73% (59.19–80.1)	80.00 (66.45–88.98)	90.91% (76.85–96.79)	96.77% (81.12–99.52)
NPV^2^ (95% CI)	97.96% (87.44–99.7)	96.36 (87.38–99.03)	100% (92.1–100)	100% (92.3–100)

^1^Positive predictive value. ^2^Negative predictive value.

## Data Availability

The data used to support the findings of this study are available from the corresponding author upon reasonable request.
